# Genetic Variability and Conservation Challenges in Lithuanian Dairy Cattle Populations

**DOI:** 10.3390/ani13223506

**Published:** 2023-11-13

**Authors:** Šarūnė Marašinskienė, Rūta Šveistienė, Violeta Razmaitė, Alma Račkauskaitė, Violeta Juškienė

**Affiliations:** Animal Science Institute, Lithuanian University of Health Sciences, R. Žebenkos 12, LT 82317 Baisogala, Lithuania; ruta.sveistiene@lsmuni.lt (R.Š.); violeta.razmaite@lsmuni.lt (V.R.); alma.rackauskaite@lsmuni.lt (A.R.); violeta.juskiene@lsmuni.lt (V.J.)

**Keywords:** Lithuanian dairy cattle, local breeds, pedigree analysis, inbreeding, effective population size

## Abstract

**Simple Summary:**

Due to the widespread adoption of commercial cattle breeds worldwide, the population size of native cattle breeds has declined dramatically. Therefore, the purpose of the present study was to investigate the genetic variability of Lithuanian cattle open populations, as well as the old genotypes, which are currently under a conservation program. The genetic variability was estimated using the number of founders, pedigree completeness, number of males and females in reproduction and age distribution, generation interval, inbreeding coefficient and effective population size. This study made it possible to compare old genotype Lithuanian dairy cattle breeds with commercial, more productive populations and highlight some traits useful in breeding programs.

**Abstract:**

The purpose of the study was to investigate the genetic variability of open Lithuanian Red and Red-and-White (LRWP) and Lithuanian Black-and-White (LBWP) dairy cattle populations and indicate the differences from the old genotypes of Lithuanian Black-and-White (LBW) and Lithuanian Red cattle (LR), which are currently under a conservation program. In order to gain a better understanding of the populations under conservation and to minimize the potential influence of other breeds, a distinct subgroup was formed that comprised animals whose father and mother belonged to the same breed (LR_pure and LBW_pure). The genetic variability was estimated using the number of founders, pedigree completeness, number of males and females in reproduction and age distribution, generation interval (GI), inbreeding coefficient (F) and effective population size (Ne). The highest average pedigree completeness values in the second generations of the old genotype LR and LBW were 100%. Higher ages of females in the populations under conservation were related to a higher GI and their longer life expectancy. In 2021, the reproductive age of bulls used for insemination within these populations ranged from 5.1 to 27.8 years. The proportions of males producing offspring in their older age indicate that the semen was used from the national gene bank of commercial artificial insemination companies. The GI (>5) in LR and LBW females was higher than that in LRWP and LBWP. The analysis of the data over the 15-year period showed that the GI of males in LRWP and LBWP decreased equally by 38%, while in LR_pure population, it increased by 80%. A high (9.24%) average inbreeding coefficient (F) was found in inbred animals of LR_pure population, while in LBW_pure, it was 5.35% in 2021. The coefficient of inbreeding varied within the different cattle populations. In the open LR population, it ranged from 1.48% to 2.7%, while in the LRWP population, it fell between 2.12% and 3.72%. The lowest effective population size (Ne) concerning the rate of inbreeding was observed in LBW_pure (23) and LR_pure (59), with the highest Ne identified in the LBWP population (462). When considering Ne based on the number of parents, LR_pure displayed the lowest Ne (42), while the highest Ne was found in LBWP (4449). An analysis of local cattle populations reveals that LR faces the most critical situation. This particular population has been steadily declining for a number of years, necessitating additional measures and efforts to safeguard the LR’s ancestral genetic makeup. The results of the LBWP analysis also highlight a concerning trend. Even in very large populations with open breeding programs, the effective population size per generation can experience a significant decrease.

## 1. Introduction

The global cattle population is highly diverse ranging from breeds specialized for very high milk production to numerous local breeds adapted to local conditions [1]. Native cattle breeds are important genetic resources considering their adjustment to the native environment in which they are bred [2]. However, due to the widespread adoption of commercial cattle breeds worldwide, the population size of native cattle breeds has declined dramatically. At the beginning of the 20th century, there were approximately 230 breeds of cattle in Western Europe, of which 70 have already become extinct, and another 53 are endangered [3]. Genetic diversity is very important from both the economic and environmental points of view, as it allows the genetic improvement of an animal’s production traits [4,5], while the effective population size (Ne) is a key parameter in population genetics. It has important applications in evolutionary biology, conservation genetics and plant and animal breeding, because it measures the rates of genetic drift and inbreeding and affects the efficacy of systematic evolutionary forces, such as mutation, selection and migration [6,7]. The level of inbreeding in dairy cattle has received attention since the first half of the last century [8,9]. The population structure determines the development of inbreeding [10], and inbreeding becomes very important when breeding small populations [11,12]. However, the accumulation of inbreeding and the loss of genetic diversity in modern dairy cattle breeds is a potential problem [9].

Dairy production is a significant agro-food industry in the European Union (EU) [13], as well as in Lithuania, where it represents approximately one-fifth of all agricultural production and holds a priority position within the agriculture sector [14]. The main goal of the dairy cattle sector is to increase the volume of milk production. In Lithuania, the data from milk recording indicate that cow productivity has increased 1.5 times from 2005 to 2021 [15]. Lithuanian Red and Red-and-White and Lithuanian Black-and-White are the main cattle populations raised in Lithuania [16]. The breeds were recognized as independent in 1951. Currently, these populations are being crossed with other breeds to increase the productivity [16]. Uncontrolled crossbreeding has threatened the existence of the original breeds. To avoid the loss of genetic resources, the most typical old genotype animals from Lithuanian Black-and-White and Lithuanian Red cattle breeds were selected and new herd books were created for the breeds’ restoration. Since 2005, the animals of Lithuanian Red cattle old genotype and Lithuanian Black-and-White old genotype have been under a conservation program and have been receiving subsidies from the Rural Development Program as critical and endangered breeds [17]. Compensatory payments have helped to conserve the genetic resources and stabilize the numbers of some Lithuanian farm animal breeds by reproducing new herds or animals following special mating rules and schemes in order to minimize inbreeding and preventing the production of highly inbred individuals. The remaining two open populations were further intensively improved. LWBP was improved with American and Canadian Holsteins, as well as Danish and German Black-and-White and British and Holstein Friesians [18], while LRWP was improved with Red-and-White Holstein, Brown Swiss and also Danish Red [19]. It is important to mention that since 1970, Denmark has sourced genetics from various red cattle breeds. These breeds include the US Brown Swiss, followed by the Red Holstein, and beginning in the 1990s, the Swedish Red and White [10]. In 2016, the number of dairy cows in Lithuania began to decrease. In total, from the beginning of 2014 to the middle of 2022, the number of dairy cows decreased by 80,486 heads. The number of registered dairy cow herds decreased, but the average herd size increased from 4.9 to 10.4 animals per herd over the period [14]. There have been a number of studies on the genetic diversity in Lithuanian dairy cattle populations based on pedigrees [19,20], microsatellite polymorphisms [21], genetic markers [22] and blood groups [23]. However, the genetic status and its changes based on the pedigree data in the two smallest old genotype critical breeds and the two largest commercial cow populations have never received overall assessment. Therefore, the purpose of the present study was to investigate the genetic variability of Lithuanian cattle in open populations (LRWP, LBWP) as well as in old genotype Lithuanian breeds (LR, LR_pure and LBW, LBW_pure).

## 2. Materials and Methods

The genetic structure was compared between two open (commercial) populations, i.e., Lithuanian Red and Red-and-White (LRWP) and Lithuanian Black-and-White (LBWP), and two old genotype populations, i.e., Lithuanian Black-and-White (LBW) and Lithuanian Red cattle (LR), which currently are under conservation. To obtain a better understanding of the populations which are under conservation and to avoid casual influence of other breeds, we have singled out a separate group of herd books (main chapter) consisting of the animals whose father and mother belong to the same breed (LR_pure and LBW_pure).

The milk performance data of Lithuanian dairy cattle breeds were collected from the productivity reports of Lithuanian Milk-recorded Cow Herds in 2020–2021 [15] and the following parameters were specified for each breed separately: milk production per year, fat (%), protein (%) and number of cows.

The data on the Lithuanian dairy cattle populations were provided by the Centre for Agricultural Information and Rural Business. The input data consisted of the unique identification of all animals: animal ID, sire ID, dam ID, birthdate and sex.

The genetic structure of the cattle populations was analyzed using the following parameters, of which (2–9) were estimated using POPREP 1.0 (http://popreport.fli.de, accessed on 12 February 2023) software system [24]:

1. The number of founders in each population at different periods: The number of reproductive males, reproductive females, founders with unknown parents, founders with only females’ known parents and founders with only males’ known parents in each period.
2. Pedigree completeness: The following formula was used to compute pedigree completeness by MacCluer et al. [25]:
  (1) $$I_{d}=4I_{dpat}I_{dmat}/I_{dpat}+I_{dmat}\;\;\;\mathrm{and}\;\;\;I_{\mathrm{dk}}=\frac{1}{d}\;\sum_{i=1}^{d}a_{i},$$
  where k represents the paternal (pat) or maternal (mat) line of an individual, a_i_ is the proportion of known ancestors in generation i, whereas d is the number of generations considered in the calculation of pedigree completeness. For example, if d = 5, then five ancestral generations will be taken into account in the computations. The values for pedigree completeness range from 0 to 1. If all ancestors of an individual to some specified generation (d) are known, then I_d_ = 1, or if one of the parents (i.e., sire or dam) is unknown, I_d_ = 0.
3. The number of males and females in reproduction by the year of offspring birth (births/select), where “births” is the number of males/females with offspring in a given year. ”Select“ represents animals born in a given year that became parents later on and determined the subset. ”Select“ represents the number of males and females represented in this subset.
4. Age distribution of males and females in reproduction by the year of birth of their offspring presents the average age of all male/ female parents.
5. Generation interval: According to Falconer and Mackay [26], the generation interval is defined as the average age of the parents at the birth of their selected offspring. It was calculated by taking the age of each of the parents at the birth of its offspring and averaging it over the age of all parents [24]. In the calculation of generation interval, an offspring is considered selected if it has produced at least one progeny. The generation intervals of males and females in the pedigree were calculated for each respective breed.
6. The inbreeding coefficient was calculated according to Wright’s [27] formula:
  (2) $$F_{X}=\sum \left[{\left(\frac{1}{2}\right)}^{n+\;n^{\prime }+1}\left(1+F_{a}\right)\right]\;$$
  where F_x_ is the inbreeding coefficient of the animal in question; n and n′ represent the number of generations between the sire and dam, respectively, and their common ancestors; and F_a_ is the inbreeding coefficient of the ancestor common to both the sire and the dam.
7. This study presents four methods for estimating the effective population size (Ne). For details, see Groeneveld et al. [24,28] and Gutiérrez et al. [29]. Based on the rates computed, the Ne is estimated as Ne = 1/2 × ∆F* for the pedigree-based methods (Table 1):

## 3. Results

### 3.1. Number of Cows and Milk Performance Data

Tables S1 and S2 depict the changes in the populations of various cattle breeds since the year 2000. As of 2021, the Lithuanian Red and Red-and-White cows (LRWP) constituted 26% of the total dairy cow population in Lithuania, with Lithuanian Red representing the majority of the Lithuanian Red and Red-and-White population at 83.99%. Following these were Ayrshire at 5.34%, Holstein at 4.12%, Swedish Red-and-White at 2.73% and other dairy breeds at 3.82%. In contrast, the Lithuanian Black-and-White population (LBWP) accounted for 73% of the total, with the majority (63.26%) being Holsteinized Lithuanian Black-and-White cows, followed by Holstein at 35.74% and other breeds at 1%. However, the two remaining breeds under the conservation program, namely the Lithuanian Black-and-White old genotype (LBW) and Lithuanian Red old genotype cattle (LR), make up a minimal 0.08% and 0.04% of the overall number of dairy cows, respectively.

Milk performance data were collected from the productivity reports of Milk Recorded Cow Herds [15] in 2020–2021 and are shown in Table 2. The analysis of the milk performance data indicated that the highest average milk productivity was found for LRWP with 8651 kg per standard lactation and the lowest for LBW with 7004 kg. However, it should be underlined that LR and LBW average milk productivity in the main herd book section is very low (LR—5440 kg, LBW—6344 kg) because only purebreds are recorded in this section.

### 3.2. Number of Founders

The analysis of Lithuanian dairy cattle pedigree records showed that in the last period (2016–2020), a higher number of founders with unknown parents (N1) was present in LBWP. Quite a large number of founders with only female’s known parents (N2) was present in LBW even in the recent period when the conservation program was approved (Table S3).

### 3.3. Composition of Pedigree, Pedigree Completeness and Generation Intervals

The composition of pedigree records and pedigree completeness is shown in Table 3. All animals, including parents, grandparents and all known ancestors, were born between 1944 and 2022.

Upon analysing the total data set for average pedigree completeness (%) for one to six generations deep by year, the average pedigree completeness for the first generation of ancestors was determined to be 100% in all six breeds, except for LR (93.8%). The average pedigree completeness values for the second and third generations were from 86.4% to 100%. As expected, the average pedigree completeness for the following generations progressively decreased; however, the absolute levels varied. The average pedigree completeness values for the fourth and fifth generations were 90.0 and 83.0%, respectively. The lowest average pedigree completeness in the sixth generation was found in LR_pure and LBW.

Table 4 presents the number of males and females in reproduction by the year of offspring birth (births/select). In the last sixteen years, the number of birth males for modern populations differ from each other. In LRWP, the number of males (birth) increased by 33%, while in LBWP, it decreased by 24%. The number of females used to produce offspring increased by 63% for LRWP and even 5.3-times more for LBWP during the analysed years (2005–2021). The analysis of the number of males in reproduction by the year of offspring birth in the populations under the conservation program showed a different situation over the sixteen-year period: LR_pure and LBW_pure decreased by 58 and 67%, respectively, while LBW increased by 31%. The analysis of the females used to produce offspring showed a tendency to increase in LRWP, LBWP, LBW and LBW_pure. The lowest number of females selected to produce their female offspring for the next generation was found in the purebred LR population and accounted for only 46% in 2005–2015, while the number in pure LBW amounted to 70%. The selection in open populations was higher and averaged 84% in LBWP.

The information about the average age distribution of males and females in reproduction by the year of their offspring birth is presented in Table 5. In 2021, the reproductive age of the bulls used for insemination of the females within these populations ranged from 5.1 to 27.8 years. The maximum average age of males used for reproduction was found in LR_pure, i.e., as much as two-times higher than that in LR. In LBW_pure, it was 8.5 and in LBW, 5.1 years. From 2005 to 2021, the average age of females in LRWP, LBWP, LR_pure and LR decreased by 20, 14, 26 and 35%, respectively, whereas in LBW_pure and LBW, it increased by 18 and 7%, respectively.

The generation interval by the year of birth and gender of each breed are presented in Table 6. The average generation intervals in the present study differed within each breed. The analysis of the 15-year results in open populations indicated that the generation interval of the male in LRWP and LBWP decreased uniformly by 38%, while that of the females decreased by 26 and 19%, respectively.

Across the years included in this study, the biggest positive change was found in LR and LR_pure breeds, where the generation interval of the males increased by 13 and 80%, respectively. The generation interval of the males for LBW and LBW_pure breeds from 2005 to 2015 decreased by 50 and 46%, respectively.

### 3.4. Inbreeding

Shown in Table 7 are the number of inbred animals and the average inbreeding coefficients in the open and closed populations.

The average inbreeding coefficient (F) in 2021 showed that the estimated inbreeding level (of all animals by year) in LR_pure was 2.4% higher than in LR, whereas in LBW_pure, it was 0.91% higher than in LBW. The estimated inbreeding level in 2021 in LBWP was 1% higher than in LRWP. The highest inbreeding coefficient was found in LBW_pure and was slightly lower in LR_pure and the lowest in LRWP and LBWP. The average estimated inbreeding coefficient indicates that inbreeding is increasing in all populations. Due to a very small number of individuals available in the population, the average inbreeding coefficient (F of inbred animals by year) in breeding the old genotype cattle in 2021 was 9.2 in LR_pure and 5.35% in LBW_pure populations. These results show that small closed populations are prone to uncontrolled increase of inbreeding, which is difficult to avoid.

### 3.5. Effective Population Size

Table 8 shows the results of the analyses using two methods to determine the effective population size (Ne) based on pedigree data: efficiency index based on the rate of inbreeding and on the number of parents. The differences between those two methods can be seen in all the analysed breeds. The highest effective population size (Ne) based on the rate of inbreeding in 2021 was found in LBWP (462), followed by LR with 103, which is 4.5-times lower than that of LBWP, and the lowest were those of LRWP (68), LR_pure (59), LBW and LBW_pure (23). In this analysis, Ne based on the number of parents differs between all the breeds. The highest Ne was estimated for LBWP, followed by LRWP.

It is worth noting that the effective population size (Ne) of LBWP decreased by 28% over the period from 2005 to 2021, while the Ne of LRWP increased by 35%. When we conducted an Ne analysis based on the number of parents for the old local dairy breeds, we found that in 2021, LBW had the highest Ne with 633 individuals. In the same year, LBW_pure had an estimated Ne of 163, while LR_pure and LR had the lowest Ne values of 107 and 42, respectively. An examination of changes over the years under study revealed that Ne estimates for LR, LR_pure and LBW decreased by 70%, 35% and 47%, respectively. However, the Ne estimates for LBW, one of the breeds under conservation, increased by 26% from 2005 to 2021. Although some of the effective population sizes were negative according to Table 8, it is possible as a result of immigrants into the population [24].

At the end of 2021, the effective population size, based on the Ne-Coan (Table 9), was calculated for LR_pure, LR, LBW and LRWP and was found to be 10, 29, 28 and 121, respectively.

The effective population size based on the Ne-Ecg method for all breeds is presented in Table 10. In 2017–2021, the effective population size based on regression on equivalent generations decreased in all the breeds. The highest decrease (71%) was found in LR_pure and the lowest (20%) in LBWP.

## 4. Discussion

Demographic data play a vital role in assessing the risk status of livestock breeds, which is a critical step in strategically planning the effective management of farm animal genetic resources [31]. The cattle selected for the conservation program, like old genotype animals from Lithuanian Red and Lithuanian Black-and-White populations, were entered into the herd book together with other animals from LBWP and LRWP until 2007. This prevented proper monitoring of animals with lower income than international breeds. Therefore, animals with old genotypes were separated and marked by different breed codes. With the aim of getting a clearer view of the conservation work, in 2019, new breeding programs were approved with stringent requirements for pure breeding, aimed at providing a clearer perspective on the conservation efforts. Consequently, the primary section of the herd book presented vital data essential for analysing individual breeds. In connection with the open population of Lithuanian Black-and-White cattle, in 2021, a decision was made that cattle with more than 75% Holstein would be attributed to Holstein population. Therefore, the number of Holsteins in Lithuania increased and that of Lithuanian Black-and-Whites decreased.

Income generated from the production of native cattle breeds is not competitive when compared to that of commercial breeds and falls short of meeting the demands of both farmers and the market. The milk performance data showed that the productivity of LRWP in the main section of the herd book per standard lactation was 7751 kg, i.e., 2135 kg less compared with Red-and-White Holsteins. The productivity of LBWP amounted to 8335 kg milk, and this was 1321 kg less compared with Black-and-White Holsteins also bred in Lithuania. Significantly lower milk production results in the main herd book were found for pure populations under the conservation program: LR_pure with 5440 kg and LBW with 6344 kg. According to Paura et al. [32], the milk productivity of the local Latvian Brown (LB) population is very similar (5302 kg per standard lactation) to the old genotype Lithuanian Red (LR) cows. Significantly lower milk yields of local breeds are partly counterbalanced by advantages in longevity, fertility and health traits [33]. Other authors have also highlighted additional significant differences of morphological traits between the breeds [34,35]. A previous study [36], in which the economic values (EVs) of functional traits for the three Lithuanian dairy cattle breeds were derived, indicated that the economic values of energy corrected milk (ECM) were 0.16 €/kg for Lithuanian Black-and-White open population (LBW) and Lithuanian Red open population (LR) and 0.21€/kg for Lithuanian Red old genotype (LROG). The higher EV for LROG was caused by a lower feed intake and a lower milk yield, compared with LR and LBW, as it was assumed that a larger proportion of concentrated feed was needed to ensure the increased milk yield [36]. In practice, environmental and economic factors, such as poor housing system, inadequate feeding, heat stress and low milk prices, play a key role in the survival of the herd and influence the length of the productive life of cows [37,38].

The productivity reports of Lithuanian Controlled Cow Herds [39] indicate that until 2000, there were so-called Red cattle. After 2000, this breed was renamed Red, Red and White. The lowest average pedigree completeness was found for the old LR and LBW populations. This could be explained by the permission to enter crossbred animals or animals without full pedigree into a traditional part of the herd book [40]. Meanwhile, when analysing only purebred cattle, the average completeness of pedigree across all six generations was determined to be higher for LR_pure with 89.0% and LBW_pure with 94.0%. Paura et al. [32] analysed two local Latvian breeds and determined similar pedigree completeness for Latvian Brown (LB) (94.23%) and Latvian Blue (LZ) (90.7%). These results were influenced by a long history of recording LB animals and by a relatively short period of LZ registration as a breed. In a study focused on endangered Spanish breeds, a lower pedigree completeness level of 82.76% was observed in ‘Berrenda en Negro’ and 79.57% in ‘Berrenda en Colorado’ [41]. According to Addo et al. [42], the estimated pedigree completeness showed that within the local German breeds, the Angler pedigree was approximately 90.0% complete, which was higher than that of the Red-and-White dual-purpose cattle in the first parental generation. The knowledge of pedigree completeness is very important, because the level of inbreeding within a breed is dependent upon the pedigree completeness of that breed and a large fraction of missing parents in a pedigree may cause serious underestimation of the inbreeding level and the associated losses arising from inbreeding [43,44].

The results of the present study showed that the average generation completeness of LRWP in six generations was higher (90%) than that of Spanish cattle, but lower than that of Latvian Brown cattle. During 2005–2021, the number of males and females used for open populations tended to increase. A long period between males producing their offspring for a new generation and a 10–16-year generation interval in both populations under the conservation program indicate that the semen from the old approved bulls in the gene bank was used for breed restoration. The data of the generation interval from open populations differ because of their intensive breeding. Lithuanian old genotype breeds have a very low number of males and females used to produce offspring and these results generally agree with the earlier analysis of local cattle populations, which showed that the old genotype Red cattle maintained in one herd at the LUHS Institute of Animal Science are in the most critical condition [17]. Activities on LBW restoration began in 2009, when eight bulls (the semen of which was still kept at breeding institutions) were selected for the breeding and conservation program.

The comparison of the populations under different breeding programs showed that the average reproductive age of females and males is higher in the populations under conservation. This can be explained by the use of artificial insemination, as semen can be used for many years after it is collected, thereby causing a large average age of LR males. The use of artificial insemination means that bulls can still be used, as their semen is preserved for many years, thereby eventually causing a large reproductive age. A higher age of females in the populations under conservation is probably related to the fact that the longevity of these cows is greater than that of highly productive cows [37]. According to Buonaiuto et al. [35], the genetic improvement of only milk production characteristics has led to the deterioration of functional traits, such as longevity and fertility. Therefore, nowadays, the breeding objective includes economically relevant traits that are not directly related to milk productivity. The dual-purpose Simmental cows with an intermediate body condition score (BCS)/muscularity are more likely to stay in the herds after the third lactation compared to those presenting a lower BCS/muscularity. This ability of cows to stay in the herd longer can be associated with their intermediate BCS/muscularity [34]. According to Adamczyk [37], cows that have lower milk yields (including the cows belonging to the so-called local breeds) are usually characterized by higher longevity. The proportions of males producing offspring in their older age indicate that males are used in reproduction longer than females. Paura et al. [32] stated that the generation interval of the male is influenced by the AI age of the male. This was also reflected in our study, and the results suggest that the generation interval is influenced by age. Since 2010, the average age of active AI bulls of the Latvian Brown breed has ranged between 13.3 and 24.6 years, and the generation interval of males ranged from 14.0 to 20.8 years. In our case, the trend was repeatedly confirmed that the age of males and females is influenced by the generation interval calculated using the average ages of the male and female parents. Sorenson et al. [10] reported that the generation interval of Danish Red was 5.0 (1997–1998) and 4.8 (2001–2003) years and of Danish Holstein, 5.0 (1983–1992) and 4.6 (1993–2003) years. A higher generation interval (6.66 years) was estimated in Irish Holstein Friesian in 2004 [42], while in our case, the generation interval for LBWP female was on average 4 years and similar to LRWP. However, the average generation interval in LR and LBW cattle populations, which are under the breed restoration program, is more than 5 years (2005–2015).

Due to a very small number of individuals available in the populations, the average inbreeding coefficient (F) of inbred animals in 2021 in the LR_pure cattle population was very high. Therefore, the work in small, closed populations demanded individual selection of pairs. The situation is better in large open populations, but even in these populations, special different breeding programs are applied. The inbreeding coefficient in the open Lithuanian Red cattle population varies from 1.48 to 2.7% and in the Holstein population, from 2.12 to 3.72%. Similar data were reported by Addo et. al. [42] in German breeds. The inbreeding coefficient of inbred individuals in German Angler (RVA) was 2.19%, and in Red-and-White dual-purpose (RDN) cattle population, it was 1.94%. Our study showed that the number of inbred individuals was lower in the populations under conservation. Inbred individuals in LR_pure and LBW_pure groups accounted for 40 and 75%, respectively, while in open populations, more than 98% of the animals were inbred. In a study by Addo et al. [42], it was found that 64% of individuals in the RVA breed and 21% in the RDN breed exhibited inbreeding. This can be attributed to the fact that larger populations tend to experience uncontrolled increases in inbreeding due to the higher priority given to increasing productivity. The need to manage inbreeding in closed populations of animals such as domestic pets, captive populations of wildlife or farmed livestock has been further emerging in international policy through individual national efforts as well as guidance from regulatory bodies such as the United Nations Farm Animal Organization [42].

The effective population size (Ne) is one of the most commonly used indicators of genetic diversity, and for animal breed conservation purposes, it can be defined in various ways [24,29,45,46]. The effective population size based on the inbreeding rate of the LBW population did not reach the recommended minimum Ne (50–100) [47]. The high inbreeding levels were influenced by an 87% decrease in the number of males in reproduction in the period between 1991 and 2020. The Ne determined for the populations analysed by Sorensen et al. [10] were 49, 53 and 47 for Holstein, Jersey and Danish Red, respectively. More recent findings are based on the studies by Paura et al. [32], who indicated that in 2015–2019, the effective population size based on inbreeding was 112 for the Latvian Brown breed. The higher the effective population size, the lower is the loss of diversity and inbreeding increase with negative consequences. The current recommendation of the United Nations Farm Animal Organization (FAO) is to maintain breeds with a maximum rate of accumulation of 1% inbreeding per generation. In order to achieve this, it is necessary to maintain a minimum Ne of 50 animals (the rate of inbreeding ΔF = 1/2Ne) [28]. In 2021, the highest effective population size (Ne (based on the number of parents) was determined in LBWP (4449) and LRWP (3855) and the lowest in LR_pure (42). This is in agreement with the authors who demonstrated that breed formation could reduce the effective population size [1]. According to Paura et al. [32], Ne based on the parents for the Latvian Brown breed was 186. The effective population size, estimated using the Ne-Coan method, exhibited a decrease across all the breeds, and in 2017–2021, the most substantial accounting of 81% was registered in LR_pure and LR breeds, while the lowest 3% decrease was found in LBWP. Similarly, estimates based on the Ne-Ecg method also demonstrated a decrease in effective population size in all the breeds, with the most significant decreases observed in the LR_pure and LR breeds, amounting to 71% and 63%, respectively. The lowest decrease (20%) in effective population size was observed in the LBWP. This study illustrates that the current breeding strategies can result in the depletion of genetic diversity in both small and large populations of cattle.

Due to the successful implementation of the conservation program, the LBW population became more consolidated and considerably more herds were established. However, the strategy of LR conservation is based on animal concentration in one conservation herd and imposes a higher risk of genetic diversity loss compared to the increased fragmentation of the population. Moreover, small closed populations are prone to inbreeding increase due to a small number of bulls and their semen availability in AI centres. Historical data show that subsidies given for the conservation program of the animals included in the herd book together with not separating crossbred cows from purebred native cows leads to a lower rate of inbreeding and prevents the loss of genetic diversity due to genetic drift.

## 5. Conclusions

Lithuanian native cattle breeds have been declining to a critical level for a number of years. The analysis of milk performance showed that milk productivity is lower in both old genotype cattle breeds in comparison with the respective breeds in open populations. It is important to note that though pure old genotype LR and LBW cattle demonstrate significantly lower productivity, their longevity is much higher. Lithuanian Red and Lithuanian Black-and-White old genotype cattle exhibited lower pedigree completeness, higher inbreeding levels and lower effective population size compared with open populations. Old genotype animals, recorded in the main section of herd books, demonstrated higher pedigree completeness compared with their populations. These results have been achieved due to the impact of the current genetic resource conservation and selection program. Even in very large populations with an open breeding program, the effective population size per generation can decrease drastically.

The analysis of local cattle populations revealed that the LR breed remains in critical condition in terms of small numbers. As a result, conservation programs are of utmost importance and should be continued with a strong emphasis on improving pedigree completeness and effective population size, managing the inbreeding coefficient and optimizing the generation interval.

## Figures and Tables

**Table 1:** Methods for estimating effective population size from PopRep.

Methods	Cascade	Formula	Description
Ne-_∆_F_p_	Animals and their parents born in generation t	∆F_p_ = (F_t_ − F_t−1_)/(1 − F_t−1_)	F_t_ = 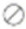 inbreeding coefficient of offspring, F_t−1_ = 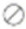 inbreeding coefficient of direct parents [26]
Ne-Cens	Parents of animals born in generation t	Ne = 4N_m_ × N_f_/(N_m_ + N_f_) × 0.7	N_m_ = number of males per generation, N_f_ = number of females per generation [30],
Ne-Coan	Animals born in generation t + 1 and t	∆f_g_ = (f_t_ − f_t−1_)/(1 − f_t−1_)	f_t_ = additive genetic relationship (AGR), f_t−1_ = 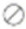 AGR of parents [26]
Ne-Ecg	Animals with their complete ancestors born in generation t	∆F_i_ = 1 − $\sqrt[{{\mathrm{Ecg}}_{i}-1}]{1-F_{i}}$	Ecg = sum of all known ancestors with (½)^n^, F_i_ = individual inbreeding coefficient [28]

Ne-∆Fp—based on the rate of inbreeding; Ne-Cens—based on the number of parents; Ne-Coan—based on the co-ancestry; Ne-Ecg—based on the regression on equivalent generations. Cascade—PopRep cascade for determining effective population size.

**Table 2:** Number of cows and milk production per year in Lithuanian dairy breeds (2021).

Breed	Production Data	Milk, kg	Fat, %	Protein, %	Number of Cows
LRWP	Herd book	8651	4.32	3.5	34,256
LBWP	Herd book	8284	4.34	3.41	94,272
LR	Herd book	8330	4.60	3.60	50
LR_pure	in main section *	5440	4.19	3.49	10
LBW	Herd book	7004	4.49	3.42	995
LBW_pure	in main section *	6344	4.41	3.38	564

* Mother and father are from the same breed.

**Table 3:** Composition of pedigree records and pedigree completeness index (PCI) for the last birth year (2021).

Breeds	Time Period	Number of Animals in Pedigree	Pedigree Completeness Index					
			PCI1	PCI2	PCI3	PCI4	PCI5	PCI6
LRWP	1946–2021	313,214	1.0	0.944	0.908	0.881	0.841	0.8
LBWP	1944–2021	354,201	1.0	0.967	0.946	0.932	0.908	0.88
LR	1959–2022	1266	0.938	0.938	0.925	0.904	0.818	0.725
LR_pure	1959–2021	974	1.0	1.0	0.962	0.902	0.79	0.684
LBW	1961–2022	9058	1.0	0.918	0.864	0.814	0.758	0.693
LBW_pure	1961–2022	5260	1.0	1.0	0.980	0.942	0.89	0.815

LRWP—Lithuanian Red and Red-and-White; LR—all old genotype Lithuanian Red cattle; LR_pure—only purebred cattle included in the main section of herd book; LBWP—Lithuanian Black-and-White; LBW—all old genotype Lithuanian Black-and-White cattle; LBW_pure—only purebred cattle included in the main section of herd book. PCI 1–6 = PCI for pedigree depths of 1 to 6 generations.

**Table 4:** The number of males and females in reproduction by year of offspring birth (birth/selected).

Year	Breed					
	LRWP	LBWP	LR	LR_pure	LBW	LBW_pure
Male						
2005	382/354	908/831	19/17	12/10	61/60	33/31
2009	388/359	764/706	10/10	4/4	47/45	24/23
2015	517/457	789/738	18/15	12/7	94/76	30/14
2020	528/183	670/295	9/4	3/3	71/13	9/4
2021	508/-	694/-	14/-	5/-	80/-	11/-
Female						
2005	11,279/6298	7498/6795	41/20	32/11	167/161	105/95
2009	9945/6145	9751/8575	15/12	9/6	216/170	140/107
2015	12,255/7175	17,623/13132	32/21	20/8	479/287	245/108
2020	16,889/1468	31,417/3367	36/6	10/3	498/27	183/6
2021	18,338/-	39,804/-	45/-	15/-	537/-	130/-

LRWP—Lithuanian Red and Red-and-White; LR—all old genotype Lithuanian Red cattle; LR_pure—only purebred cattle included in the main section of herd book; LBWP—Lithuanian Black-and-White; LBW—all old genotype Lithuanian Black-and-White cattle; LBW_pure—only purebred cattle included in the main section of herd book. In each column, the first number shows the number of animals born, and the second number represents the number of animals selected to produce offspring.

**Table 5:** Average age distribution of males and females in reproduction by year of their offspring birth.

	Year	LRWP	LBWP	LR	LR_pure	LBW	LBW_pure
Male	2005	7.7	6.0	9.4	9.5	7.3	8.3
	2009	8.0	6.6	11.5	14.0	7.7	8.3
	2015	6.5	5.6	8.7	10.9	4.9	7.5
	2020	5.7	5.2	14.7	31.0	5.6	7.9
	2021	5.5	5.2	13.0	27.8	5.1	8.5
Female	2005	4.1	3.7	5.1	5.0	4.2	4.4
	2009	3.9	3.7	7.3	7.2	5.3	6.0
	2015	3.5	3.4	3.4	3.5	3.7	4.1
	2020	3.3	3.2	3.8	4.5	4.4	4.9
	2021	3.3	3.2	3.3	3.7	4.5	5.2

LRWP—Lithuanian Red and Red-and-White; LR—all old genotype Lithuanian Red cattle; LR_pure—only purebred cattle included in the main section of herd book; LBWP—Lithuanian Black-and-White; LBW—all old genotype Lithuanian Black-and-White cattle; LBW_pure—only purebred cattle included in the main section of herd book.

**Table 6:** Generation interval distribution of males and females.

	Year	LRWP	LBWP	LR	LR_pure	LBW	LBW_pure
Male	2005	9.4	8.2	8.9	8.6	9.3	10.1
	2009	8.5	8.7	14.2	16.6	9.7	10.7
	2015	7.8	7.4	10.1	15.5	4.7	5.5
	2020	5.8	5.1	-	-	-	-
Female	2005	4.6	4.2	4.4	4.0	4.6	4.7
	2009	4.4	4.3	7.8	7.7	5.6	6.3
	2015	4.0	4.0	3.8	4.0	3.8	4.5
	2020	3.4	3.4	-	-	-	-

LRWP—Lithuanian Red and Red-and-White; LR—all old genotype Lithuanian Red cattle; LR_pure—only purebred cattle included in the main section of herd book; LBWP—Lithuanian Black-and-White; LBW—all old genotype Lithuanian Black-and-White cattle; LBW_pure—only purebred cattle included in the main section of herd book.

**Table 7:** Number of inbred animals and average inbreeding coefficient (2005–2021).

	Year	LRWP		LBWP		LR		LR_pure		LBW		LBW_pure	
		N	F	N	F	N	F	N	F	N	F	N	F
F of all animals by year	2005	11,541	0.0098	7726	0.0143	41	0.0008	32	0.0010	170	0.0044	170	0.0042
	2009	10,097	0.0105	9972	0.0191	15	0.0333	9	0.0556	221	0.0065	143	0.0061
	2015	12,510	0.0173	18,011	0.0266	33	0.0055	20	0.0088	489	0.0187	249	0.0171
	2020	17,441	0.0269	32,470	0.0352	38	0.0045	10	0.0018	516	0.0336	188	0.0349
	2021	19,010	0.0265	41,226	0.0369	48	0.0129	15	0.0370	551	0.0416	135	0.0507
F of inbred animals by year	2005	6463	0.0175	5235	0.0212	1	0.0313	1	0.0313	43	0.0173	30	0.0148
	2009	7187	0.0148	8417	0.0226	2	0.2500	2	0.2500	73	0.0198	48	0.0181
	2015	11,141	0.0194	17,088	0.0280	6	0.0304	4	0.0439	393	0.0233	215	0.0198
	2020	16,773	0.0280	32,046	0.0357	12	0.0142	1	0.0176	465	0.0373	181	0.0362
	2021	18,595	0.0271	40,820	0.0372	26	0.0238	6	0.0924	504	0.0455	128	0.0535

LRWP—Lithuanian Red and Red-and-White; LR—all old genotype Lithuanian Red cattle; LR_pure—only purebred cattle included in the main section of herd book; LBWP—Lithuanian Black-and-White; LBW—all old genotype Lithuanian Black-and-White cattle; LBW_pure—only purebred cattle included in the main section of herd book. N—number of inbred animals; F—average inbreeding coefficient.

**Table 8:** Effective population size based on the rate of inbreeding Ne (∆F) and number of parents Ne.

		LRWP	LBWP	LR	LR_pure	LBW	LBW_pure
Ne (∆F)	2005	90	-	-	−1158	171	100
	2009	-	-	195	168	659	219
	2015	100	217	253	95	46	42
	2020	67	211	106	70	25	27
	2021	68	462	103	59	23	23
Ne	2005	2857	6209	164	138	468	305
	2009	2985	5585	156	107	442	298
	2015	3954	4962	150	71	746	342
	2020	3923	4640	117	50	659	204
	2021	3855	4449	107	42	633	163

LRWP—Lithuanian Red and Red-and-White; LR—all old genotype Lithuanian Red cattle; LR_pure—only purebred cattle included in the main section of herd book; LBWP—Lithuanian Black-and-White; LBW—all old genotype Lithuanian Black-and-White cattle; LBW_pure—only purebred cattle included in the main section of herd book; Ne (∆F)—effective population size based on the rate of inbreeding; Ne—effective population size based on the number of parents; -—negative values.

**Table 9:** Effective population size based on Ne-Coan method.

Years	LRWP *	LBWP	LR *	LR_pure *	LBW *	LBW_pure
2017	231	60	155	54	125	34
2018	142	45	118	35	53	15
2019	120	41	71	22	33	12
2020	120	43	42	13	30	10
2021	121	58	29	10	28	10
Data history	2018–2027	2019–2026	2016–2029	2016–2029	2018–2027	2017–2028

LRWP—Lithuanian Red and Red-and-White; LR—all old genotype Lithuanian Red cattle; LR_pure—only purebred cattle included in the main section of herd book; LBWP—Lithuanian Black-and-White; LBW—all old genotype Lithuanian Black-and-White cattle; LBW_pure—only purebred cattle included in the main section of herd book. * Proposed based on the PopRep program.

**Table 10:** Effective population size based on the Ne-Ecg method.

Years	LRWP	LBWP	LR	LR_pure	LBW	LBW_pure
2017	67	98	457	277	56	73
2018	59	93	280	181	47	68
2019	54	89	219	128	38	64
2020	48	83	224	124	34	60
2021	44	79	168	79	31	53
Data history	1946–2022	1944–2022	1959–2022	1959–2022	1961–2022	1961–2022

LRWP—Lithuanian Red and Red-and-White; LR—all old genotype Lithuanian Red cattle; LR_pure—only purebred cattle included in the main section of herd book; LBWP—Lithuanian Black-and-White; LBW—all old genotype Lithuanian Black-and-White cattle; LBW_pure—only purebred cattle included in the main section of herd book.

## Data Availability

The results are available within the article, but the original raw data are not provided in the manuscript.

## Supplementary Materials

The following supporting information can be downloaded at: https://www.mdpi.com/article/10.3390/ani13223506/s1. Table S1. Annual counts of various cow breeds in Lithuanian Red and Red-and-White Cattle (2000–2021), Table S2. Annual counts of various cow breeds in Lithuanian Black-and-White Cattle (2000–2021), Table S3. The number of founders in Lithuanian dairy cattle breeds in different time periods.
