# Supplementary material for: Genetic Variability and Conservation Challenges in Lithuanian Dairy Cattle Populations
Source: Animals (Basel). 2023 Nov 13;13(22):3506. doi: 10.3390/ani13223506 (PMC10668635; doi:10.3390/ani13223506)
Supplement: Supplementary file 1 [file animals-13-03506-s001.zip › animals-2521069-supplementary.pdf]

Supplementary Materials: Table S1, Table S2, Table S3

Genetic Variability and Conservation Challenges in Lithuanian Dairy Cattle Populations

Šarūnė Marašinskienė *, Rūta Šveistienė, Violeta Razmaitė, Alma Račkauskaitė and Violeta Juškienė

Animal Science Institute, Lithuanian University of Health Sciences, R. Žebenkos 12, LT 82317 Baisogala, Lithuania; ruta.sveistiene@lsmuni.lt (R.Š.); violeta.razmaite@lsmuni.lt (V.R.); alma.rackauskaite@lsmuni.lt (A.R.); violeta.juskiene@lsmuni.lt (V.J.)
* Correspondence: sarune.marasinskiene@lsmuni.lt

Table S1: Annual Counts of Various Cow Breeds in Lithuanian Red and Red-and-White Cattle (2000-2021).

	Year						
**Breeds**	**2000**	**2005**	**2008**	**2010**	**2016**	**2020**	**2021**
LR open population	28914	46912	38921	31738	30866	29845	28772
Angler	194	254	555	480	440	472	474
Danish	202	296	610	532	483	481	460
Ayrshire	312	511	1446	1603	1567	1881	1829
Brown Swiss	39	43	55	50	26	14	13
German R&W	209	334	417	357	212	176	179
Swedish R&W	248	454	776	763	936	914	936
Holstein		105	742	660	766	1228	1412
Estonian			30	27	10	7	9
Simmental			143	189	111	144	141
Norwegian				1	17	1	1
Latvian			1		12	10	11
Dutch R&W					6	20	18
LR old genotype				92	40	53	50

Table S2: Annual Counts of Various Cow Breeds in Lithuanian Black-and-White Cattle (2000-2021).

	Year						
**Breeds**	**2000**	**2005**	**2008**	**2010**	**2016**	**2020**	**2021**
LBW open population	64629	151312	131771	103739	87097	64612	59635
Holstein	1056	1363	4321	4085	17453	30172	33694
German	1180	1339	1370	1119	720	513	460
British Fr.	74	80	101	92	87	46	36
Dutch BW	41	65	182	161	112	103	101
Danish BW	190	242	460	519	372	228	209
Swedish BW	63	253	309	341	250	150	136
LBW old genotype			100	463	932	1011	995

Table S3: The number of founders in Lithuanian dairy cattle breeds in different time periods

Periods	Male	Female	N_1	N_2	N_3
**LRWP**					
1946-2004	9914	204897	13409	7694	38386
2005-2009	2087	115203	85	4613	69
2010-2015	2562	142771	3	13023	27
2016-2020	12411	93109	-	11927	-
**LBWP**					
1944-2004	13656	68490	6365	1467	6304
2005-2009	3627	81988	740	1882	273
2010-2015	4170	170877	884	8022	214
2016-2020	23988	155363	467	12327	3
**LR**					
1959-2004	626	1521	2	-	248
2005-2009	104	279	-	-	-
2010-2015	145	423	-	5	-
2016-2020	45	310	-	13	-
**LR_pure**					
1959-2004	275	751	1	-	121
2005-2009	20	128	-	-	-
2010-2015	16	130	-	-	-
2016-2020	11	59	-	-	-
**LBW**					
1966-2004	1744	5447	176	84	1672
2005-2009	395	2828	4	25	5
2010-2015	579	4499	-	344	-
2016-2020	559	2523	-	390	-
**LBW_pure**					
1961-2004	688	2512	78	40	805
2005-2009	112	1430	3	7	1
2010-2015	83	2318	-	28	-
2016-2020	122	1090	-	5	-

N_1 - founders with unknown parents;

N_2 -founders with only female‘s known parents;

N_3-founders with only male‘s known parents.
